# Factors Influencing Disease Stability and Response to Tocilizumab Therapy in Severe COVID-19: A Retrospective Cohort Study

**DOI:** 10.3390/antibiotics11081078

**Published:** 2022-08-09

**Authors:** Wael Hafez, Ahmed Abdelrahman

**Affiliations:** 1 NMC Royal Hospital, Abu Dhabi P.O. BOX 764659, United Arab Emirates; 2 The Medical Research Division, Department of Internal Medicine, The National Research Centre, Cairo 12622, Egypt; 3 Internal Medicine Department, Zagazig School of Medicine, Zagazig 44519, Egypt

**Keywords:** COVID-19, SARS-CoV-2, tocilizumab, disease stability, severity, mortality, secondary infection

## Abstract

(1) Background: The efficacy of tocilizumab in COVID-19 has been doubted. The study aimed to investigate factors affecting disease stability and response to tocilizumab among severe COVID-19 patients. (2) Methods: This was a cohort study of 70 severe COVID-19 patients at NMC Royal Hospital, UAE, from April to June 2020. (3) Results: Elderly patients and those with cardiovascular comorbidities had a higher risk of unstable COVID-19 (*p* = 0.025). Regarding tocilizumab therapy timing, compared to the critical group receiving tocilizumab, the unstable severe patients receiving tocilizumab had a significantly higher rate of improvement (86%). In contrast, the late critical subgroup showed a significantly increased mortality rate (52.9%). The risk for secondary infection and adverse events following tocilizumab was higher in the late critical group than in the unstable severe and early critical groups (*p* = 0.024 and *p* = 0.006, respectively). Therapeutic doses of anticoagulation and high-dose vitamin D were correlated with better outcomes than the prophylactic dose and the treatment dose of vitamin D (*p* < 0.001 and *p* = 0.07, respectively). (4) Conclusions: elderly patients and those with cardiovascular disease developed unstable COVID-19. Tocilizumab is a potentially effective choice against severe and critical COVID-19. Early tocilizumab administration combined with therapeutic dose anticoagulation and high vitamin D doses could improve the patients’ outcomes.

## 1. Introduction

The coronavirus disease 2019 (COVID-19) outbreak, caused by the severe acute respiratory syndrome coronavirus-2 (SARS-CoV-2), has spread rapidly, with high death rates [1]. Interstitial pneumonia and respiratory failure were also the leading causes of death in COVID-19 [2].

Xu et al. [3] documented both a peripheral blood circulation cytometric study and histopathology from the lungs of COVID-19 patients. They observed elevated CD8 T cells in the blood and diffuse alveolar injury with diffuse mononuclear inflammatory infiltrates predominantly by lymphocytes. This result indicates that immune hyperactivation may be responsible for a significant portion of lung damage. High interleukin 6 (IL-6) plasma concentrations were a negative predictor of survival since mortality and lung injury were common in individuals with greater IL-6 levels [4]. 

Because the clinical severity of COVID-19 is linked to a cytokine storm and excess production of soluble inflammatory mediators, many immune-modulatory drugs are being studied [5]. IL-6 is a cytokine that has a role in the normal inflammatory and immunological responses [6]. Still, it is also identified as a primary driver in the cytokine storm linked with severe illnesses [7]. As a result, anti-IL-6 drugs could be a potential COVID-19 therapy [8].

As a result, tocilizumab (TCZ), a recombinant human-like monoclonal antibody that acts as an IL-6 receptor antagonist, has been recommended as a therapy for severe COVID-19 [9]. Few reported studies have discussed the role of TCZ in severe COVID-19. Luo and colleagues recently published a study that showed that a single dosage of TCZ did not improve disease in critically ill patients but adding a second dose enhanced the outcomes [10]. 

On the other hand, TCZ demonstrated a significant and stable response in severe COVID-19 pneumonia patients accompanied by hyperinflammatory symptoms and was also linked to considerable clinical improvement [11]. Although TCZ-treated patients had higher values of biomarkers predictive of cytokine storm, Ramaswamy et al. [12] found that TCZ offered short-term survival benefit. As a result, 21 COVID-19 cases were later managed in Wuhan with parenteral TCZ, which is thought to be beneficial. Indeed, as evidenced by a lung CT scan and SpO2, this study reported an improvement in pneumonia [13].

Hoffmann-La Roche has reported disappointing results from the phase 3 COVACTA TCZ trial [14], raising concerns about the efficacy of IL-6 blockade in severe COVID-19 pneumonia. The randomized evaluation of the COVID-19 therapy (RECOVERY) trial [15] has shown that TCZ reduced the risk of death in hospitalized patients with severe COVID-19. TCZ also minimized the hospitalization time and need for mechanical ventilation.

Given these controversies, patient characteristics, the timing of TCZ therapy, and the co-therapy used are likely factors influencing disease stability and response to TCZ. So, we investigated the parameters impacting disease stability and responsiveness to TCZ in severe COVID-19 patients.

## 2. Results

### 2.1. Demographic, Clinical, and Biochemical Characteristics of the Study Population

The study included 70 severe COVID-19 patients. The comparative analysis between the two severe COVID-19 groups (received–not received TCZ), where TCZ was administered only in severe cases with an unstable deteriorating course and was not given to severe cases with stable courses, showed that COVID-19 patients with cardiovascular diseases (CVS) significantly developed unstable cases than patients without CVS (*p* = 0.025). Unstable cases also showed decreased white blood cells (WBCs) and platelets while significantly increased D-dimer, IL-6, and ferritin levels compared to the stable cases (Table 1).

### 2.2. Association between the Demographic, Clinical, and Biochemical Characteristics and COVID-19 Outcomes

There was no statistically significant effect of TCZ on the overall COVID-19 outcome since patients who received it were 100% unstable cases (*p* = 0.994). In contrast, the mortality odds among late critical, unstable COVID-19 patients receiving TCZ increased significantly by 7-fold compared to severe cases (OR = 7.03, 95% CI: (1.80–32.32), *p* = 0.007). The mortality odds also increased significantly by 6% for each year increase in age (OR = 1.06, 95% CI: (1.01–1.13), *p* = 0.027).

The mortality odds decreased significantly among patients receiving TCZ, by 41%, 1%, and 15% for each unit increase in hemoglobin, platelets, and lymphocyte count, respectively. However, the mortality odds among patients receiving TCZ increased significantly by 1%, 14%, 11%, 86%, and 15% for each unit increase in C-reactive protein (CRP), D-dimer, neutrophil to lymphocyte ratio (NLR), red cell distribution width (RDW.CV), and neutrophil count, respectively. Patients receiving TCZ with a positive Coombs test also showed a significant increase in the mortality odds, by 5.4-fold (OR = 5.40, 95% CI: (1.13–29.64), *p* = 0.040) (Table 2).

**Figure 1:**
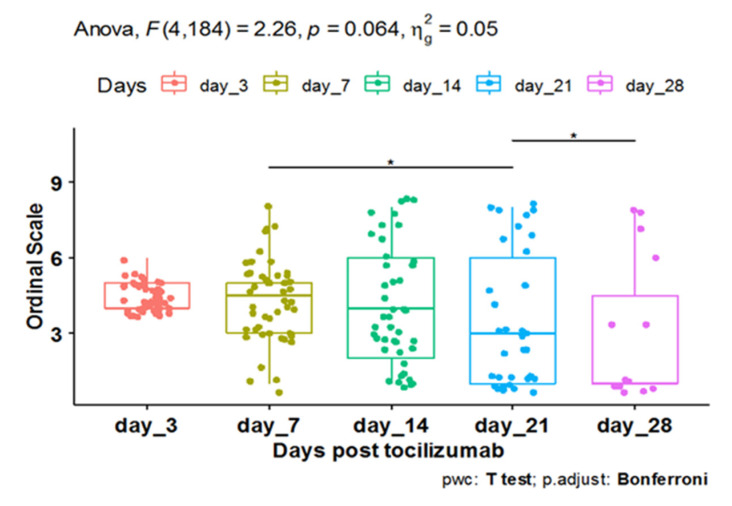
One-way Repetitive ANOVA for the comparison of the continuous WHO 8 ordinal scale at fixed time points (Days 3, 7, 14, 21, and 28) post-tocilizumab. (*: Significant Difference in the Pairwise Comparison).

The pairwise comparisons showed that day 28 post-TCZ administration had a significant decrease in the WHO scale, with a median of 1.0 and interquartile range (IQR) of 1.0–4.5, compared to day 21, with a median of 3.0 and IQR of 1.0–6.0 (adj. *p* value = 0.021). Meanwhile, the scale on day 21 showed a significant decrease compared to the scale on day 7 post-TCZ, with a median of 4.5 and IQR of 3.0–5.0 (adj. *p* value = 0.025), indicating a significant clinical improvement by reaching 28 days of the therapy (Figure 1).

### 2.3. Time to Viral Clearance among Unstable COVID-19 Patients

**Figure 2:**
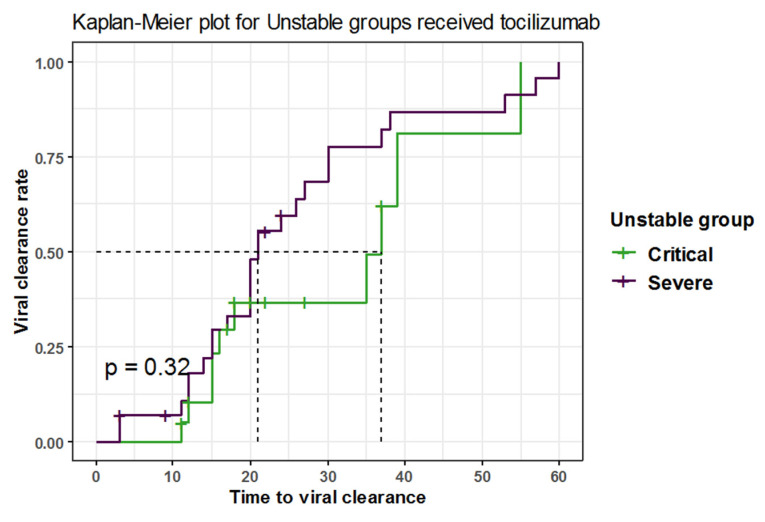
Kaplan–Meier plot for unstable groups receiving tocilizumab.

The median time to clearance in severe unstable COVID-19 patients after receiving TCZ was 21 days, with 95% CI of 20–30 days, while in critically unstable patients (early and late), it was 37 days with 95% CI of 18–NA days, showing a non-statistically significant difference (*p*-value for log-rank test = 0.3, log-rank = 1) (Figure 2).

### 2.4. The Effect of Combination of Vitamin D, Anticoagulants, Steroids, and Antivirals with Tocilizumab

Table 3 revealed that 100% of the patients with a low vitamin D status (less than 20 ng/mL) receiving high doses of vitamin D (50,000 IU every other day for two weeks or one intramuscular shot of 300,000 IU) showed clinical improvement compared to those receiving the usual treatment doses (10,000 IU daily or less) or those who did not receive it. The significance is likely hampered by the small sample size affecting the study power (*p* = 0.07). Furthermore, 100% of patients receiving non-therapeutic (prophylactic or medium) doses of anticoagulants died, and 89.7% of patients receiving therapeutic doses (1 mg/kg every 12 h) improved, indicating a significant improvement (*p* < 0.001). However, neither coadministered antivirals nor steroids significantly affected the COVID-19 outcome (*p* = 0.498 and *p* = 0.193).

### 2.5. The Rate of Adverse Events Following Tocilizumab in Different Study Groups

Table 4 showed that 60.0% and 72.7% of high-grade adverse events appeared in patients at the late critical stage during TCZ administration, and significantly higher than 90.9% of low-grade adverse events in patients were at the severe stage (*p* = 0.006). Moreover, 80.0% of secondary bacterial infections appeared in patients at the late critical stage, significantly higher than in those at the severe stage who did not develop a bacterial infection. Furthermore, 65.9% of patients who did not develop any secondary infection were at the severe stage, significantly higher than patients at the early and late critical stages (*p* = 0.024).

## 3. Discussion

In terms of TCZ therapy timing, when comparing with the group of critical patients (WHO ordinary scale of 5 or higher), the unstable severe patients (scale of 4) receiving TCZ had a significantly higher rate of improvement. In contrast, the late critical subgroup showed a significantly increased mortality rate (52.9%). The odds of mortality among the late critical subgroup of COVID-19 patients receiving TCZ increased dramatically by 7-fold. However, we recommend using it cautiously because of drug-related side effects, such as transient respiratory deterioration and secondary bacterial/fungal infections.

Although the cytopathogenic influence of the viral infection on the lung and viral evasion are essential factors in patients’ outcomes in human coronavirus infections, there is proof of a process where acute respiratory distress symptoms evolve and are maintained by overactive inflammatory stimulation [16]. It has been suggested that the SARS-CoV nucleocapsid protein could boost IL-6 production [17]. This retrospective analysis corroborates previous research that found similar processes in COVID-19 pneumonia [18]. There are currently little data on the efficacy of TCZ in COVID-19, as a few reports in specific populations have been published [19].

Guaraldi et al. [20] published a study evaluating a cohort of unselected patients receiving TCZ; the analysis included patients with less severe baseline disease characteristics. However, the reduction in mortality was comparable to our findings. Our results indicate that TCZ may be helpful in early critically ill patients. Still, it has little effect on the survival of late critical cases, even though it may delay disease progression or the need for mechanical ventilators. In agreement to our findings, the RECOVERY trial reported that the use of TCZ in invasively ventilated patients (categories 7–9) did not reduce mortality [21]. The REMAP-CAP study showed that TCZ was beneficial in invasively ventilated patients when used early within 12 h from admission [22]. Furthermore, the ECCMID has recently recommended the use of TCZ for treatment of severe COVID-19 based on moderate quality of evidence for mortality, and high quality of evidence for mechanical ventilation [23].

The plausible explanation for this significant improvement in outcome when TCZ was administered in the severe subgroup compared to the critical one is that more cytokines and inflammatory mediators are recruited with more advanced hyperinflammation, which may be too much to be aborted with TCZ, unlike early inflammation mediated by less inflammatory mediators.

In our study, the median time to viral clearance in severely unstable COVID-19 patients after receiving TCZ was 21 days, while in critically unstable patients (early and late), the median time was 37 days, showing a non-statistically significant difference. Interestingly, TCZ was linked to significantly more extended hospitalization in a previous trial. This observation could be due to various biochemical and infectious consequences [24].

Here, the odds of mortality decreased significantly among severe COVID-19 patients receiving TCZ by increased hemoglobin, platelets, and lymphocyte count. However, the odds increased significantly by increased CRP, D-dimer, neutrophil-lymphocyte ratio (NLR), red cell distribution width (RDW.CV), and neutrophil count. In a similar study, there was a transitory reduction in overall leukocytes in the initial days after using TCZ, although they eventually increased mostly due to increased lymphocytes. This apparent discrepancy could be explained by the different treatment schedules and short duration of follow-up.

On the other hand, D-dimer increased significantly despite improvements in other inflammatory markers, which could indicate a long-term coagulation problem [24]. In COVID-19, disruption of coagulative homeostasis has already been linked to death [25]. Our data indicated a substantial improvement in patients using therapeutic anticoagulants compared to prophylactic or intermediate anticoagulants (*p* < 0.001). 

A recent meta-analysis by Nugroho et al. studied the efficacy of TCZ in severe and critically ill COVID-19 patients. The authors demonstrated that TCZ reduced all-cause mortality when CRP levels were ≥100 mg/L, and the PaO2/FiO2 ratios were 200–300 mmHg or <200 mmHg. Furthermore, the subgroup analysis showed that TCZ administration in patients with CRP levels < 100 mg/L reduced neither mortality nor length of hospitalisation [26]. According to previous research, critical patients have greater levels of IL-6, which increases their chance of mortality [27]. We found a significant rise in IL-6 levels in patients with a poor outcome compared to those with a stable or better clinical status. Furthermore, this cytokine can cause a large, dose-dependent rise in respiratory resistance mechanisms [28]. IL-6 appears to play a role in lung disease progression. It is common to observe an increase in IL-6 in the first few hours after receiving TCZ; this may trigger a worsening of the inflammatory process that TCZ can not totally control in some cases. The significant drop in P/F in the lung also could be due to a greater cytokine storm sequestered. As a result, TCZ may be considered in conjunction with steroids [24].

Our data revealed no significant effect of steroid use. However, our study included patients admitted between April and June 2020, when the recovery study recommendations of steroids as a standard of care had not been published, and there was apprehension about steroids. As a result, few patients in our study received steroids with TCZ, and the consequences of our study considering steroids cannot be generalized. Additionally, in a recent systematic review, a clear benefit of combination of IL-6 antagonists and steroids were reported [29]. Furthermore, there was no statistically significant difference in the rate of clinical improvement after the coadministered antiviral therapies, indicating the efficacy of the co-administration with antiviral remains questionable.

Previous research found a link between vitamin D deficiency and COVID-19 outcome, and other studies also found that vitamin D-deficient individuals required intense oxygen therapy and hospitalization [30]. In our study, vitamin D-deficient patients receiving high doses of vitamin D (50,000 IU once every other day for 2 weeks, or 300,000 IU once orally or intramuscular injection) showed better clinical improvement compared to patients who received the usual smaller treatment doses (10,000 IU once daily) or did not receive it. Still, the significance is affected by the small sample size affecting the study power. To reduce this disparity, we need to confirm these findings in larger cohorts, assessing more incidents and considering additional potential variables such as obesity or comorbidities [31].

We found that COVID-19 patients with CVS significantly developed a more unstable state. In recent research, heart failure has been established as a potential cause of severe clinical outcomes and fatality, i.e., as a possible outcome of COVID-19-related myocardial injury [32]. In addition, the odds of unstable COVID-19 cases increased significantly by 4.9-fold in white patients compared to Asian and black ones. According to the literature, despite adjusting for all previously reported confounders, including frailty, individuals from Asian and black origins had greater mortality from COVID-19 infection [33]. This discrepancy could be related to our study’s small sample size and scope.

Here, critical patients compared to severe ones receiving TCZ had a higher rate of adverse events and secondary bacterial and/or fungal infections resulting from their immunocompromised status or the ventilation process. These findings corroborated previous research that found that the overall rate of infective complications with TCZ is considerable (70%), and the proportion of serious occurrences is low (3%). Severe infections occurred in more than 10%, indicating that these consequences are the main disadvantage of TCZ. Because the effect of TCZ on temperature and inflammatory biomarkers may delay the detection of an underlying infection, resulting in severe presentations, the active bacterial infection must be confirmed before therapy, and microbiological monitoring should be considered in the absence of apparent symptoms [24].

There are very limited data regarding TCZ safety and efficacy with respect to SARS-CoV-2 variants. A recent study showed that TCZ efficacy in severe COVID-19 does not differ between Delta or non-Delta virus variants. The Delta variant also did not increase the mortality risk when compared to other virus strains [34].

Due to sample size limitations, the study power was insufficient to investigate the effect size between the two groups, giving underestimated odds ratios for the disease severity and stability, resulting in a beta error. Additionally, the study was conducted early during the first wave of the pandemic, so our findings should be interpreted with caution because of the emergence of several SARS-CoV-2 variants that may affect the clinical course and response to different treatments.

## 4. Materials and Methods

### 4.1. Institutional Review Board IRB

The study followed the Helsinki Declaration. The Regional Research Ethics Committee, Department of Health, Abu Dhabi, United Arab Emirates (UAE), reviewed and approved the study (Ref: ADHRTC-2021-178).

### 4.2. Study Design and Study Population

This was a retrospective cohort study based on medical records of severe COVID-19 patients at NMC Royal Hospital in Khalifa City, Abu Dhabi, from 8 April 2020 to 15 June 2020. Samples were acquired using nasopharyngeal swabs and evaluated using the RT-PCR technique. According to the UAE, per the guidelines of May 2020, severe COVID-19 was defined by the presence of pneumonia plus one of the following: respiration rate of >30 breaths/minute; significant respiratory distress; or a SpO_2_ < 93% in indoor air or a need for oxygen to keep an oxygen (O_2_) saturation of over 93%. The time between the first positive RT-PCR test and the first negative of two consecutive negative RT-PCR tests was used to determine the time to viral clearance. 

Furthermore, we classified COVID-19 patients using the World Health Organization (WHO) classification criteria [35]. We classified the subjects into two groups depending on disease severity course: (1) stable severe illness (did not receive TCZ): patients in need of low flow oxygen to maintain O_2_ sat above 93%, with no significant need for higher oxygen requirements till recovery; and (2) severe unstable illness (received TCZ): patients needing oxygen to maintain O_2_ saturation above 93%, with a significant deteriorating course. Then the severe unstable illness was re-categorized into three subgroups according to the time of TCZ administration: severe unstable illness (required low flow oxygen only), early critical illness (within 24 h of needed high flow oxygen and/or noninvasive ventilation), and late critical illness (after 24 h of noninvasive ventilation requirements).

### 4.3. Data Management and Statistical Analysis

All data were statistically analyzed utilizing R Software version 3.5.2 (20 December 2018)—” Eggshell Igloo”. We used the mean ± standard deviation (±SD) for quantitative data. When the normal distribution was violated, the median and interquartile range (IQR) was applied, and the distribution was validated with a Shapiro–Wilk test. Frequency (n) and percentage (%) were used for qualitative categorical data. We utilized univariate and multivariate logistic regression models to investigate the correlation of the demographic, clinical, and biochemical characteristics. Comparative analysis between the clinical status was assessed by the WHO 8 ordinal scale and one-way repetitive ANOVA measure for comparing the WHO scale at fixed time points (Days 3, 7, and 14) and post-TCZ by Chi-square test. Kaplan Meier’s approach was used to investigate the time till clearance, and the comparison was made using a log-rank test. The *p*-value was declared significant when *p* < 0.05 with a 95% confidence interval.

## 5. Conclusions

This study supports tocilizumab’s therapeutic potential in severe and critically ill COVID-19 patients. TCZ should be initiated early, especially in patients at higher risk of unstable COVID-19. In addition, we recommend combining it with a therapeutic anticoagulation dose and high vitamin D doses in vitamin-deficient patients to improve the outcome.

## Figures and Tables

**Table 1:** Comparative analysis between COVID-19 groups (Received–Not Received) for tocilizumab regarding the demographic, clinical, and biochemical characteristics.

Baseline Characteristics		Not Received Tocilizumab N = 21	Received Tocilizumab N = 49	*p* Value
**Demographic**				
Age (years)	**Mean ± SD**	47.0 ± 9.0	50.2 ± 13.3	**0.362**
Gender	**Female**	3 (27.3%)	8 (72.7%)	**1.00**
	**Male**	18 (30.5%)	41 (69.5%)	
Race	**Asian**	19 (38.0%)	31 (62.0%)	**0.066**
	**Black**	0 (0.0%)	2 (100.0%)	
	**White**	2 (11.1%)	16 (88.9%)	
BMI	**Mean ± SD**	29.0 ±5.4	30.3 ± 6.5	**0.401**
**Clinical**				
HTN	**No**	11 (28.9%)	27 (71.1%)	**0.536**
	**Yes**	4 (18.2%)	18 (81.8%)	
DM	**No**	14 (34.1%)	27 (65.9%)	**0.804**
	**Yes**	7 (28.0%)	18 (72.0%)	
CVS	**No**	21 (38.2%)	34 (61.8%)	**0.025**
	**Yes**	0 (0.0%)	9 (100.0%)	
**Biochemical**				
WBC	**Mean ± SD**	7.6 ± 2.3	6.8 ± 3.9	**0.015**
HEMOGLOBIN	**Mean ± SD**	13.3 ± 1.6	13.1 ± 1.9	**0.682**
PLATELETS	**Mean ± SD**	427.0 ± 210.9	307.4 ± 135.2	**0.036**
CRP	**Mean ± SD**	114.0 ± 48.5	135.6 ± 107.1	**0.827**
D.DIMER	**Mean ± SD**	1.0 ± 0.5	8.9 ± 10.2	**<0.001**
IL6	**Mean ± SD**	51.4 ± 35.0	961.4 ± 1162.9	**0.03**
LDH	**Mean ± SD**	445.7 ± 141.8	641.3 ± 577.3	**0.223**
ALT	**Mean ± SD**	63.7 ± 28.3	62.7 ± 81.9	**0.058**
AST	**Mean ± SD**	70.7 ± 41.9	60.8 ± 40.7	**0.175**
CREATININE	**Mean ± SD**	0.9 ± 0.3	1.1 ± 0.9	**0.893**
NEUTROPHIL.COUNT	**Mean ± SD**	72.7 ± 13.1	75.3 ± 14.6	**0.333**
LYMPHOCYTE.COUNT	**Mean ± SD**	19.6 ± 11.7	18.1 ± 12.1	**0.53**
NLR	**Mean ± SD**	5.2 ± 3.2	7.9 ± 7.9	**0.547**
RDW.CV	**Mean ± SD**	13.0 ± 1.0	14.2 ± 2.6	**0.06**
FIBRINOGEN	**Mean ± SD**	711.5 ± 127.9	757.8 ± 170.6	**0.218**
FERRITIN	**Mean ± SD**	1284.2 ± 1905.0	1824.9 ± 1485.9	**0.001**
COOMB.TEST	**Negative**	10 (31.2%)	22 (68.8%)	**1.00**
	**Positive**	5 (31.2%)	11 (68.8%)	
ADAMTS13	**Mean ± SD**	59.5 ± 24.3	60.1 ± 15.1	**0.926**
BLOOD.GROUP	**A**	3 (20.0%)	12 (80.0%)	**0.646**
	**AB**	2 (33.3%)	4 (66.7%)	
	**B**	4 (44.4%)	5 (55.6%)	
	**O**	4 (28.6%)	10 (71.4%)	
RH	**Negative**	2 (40.0%)	3 (60.0%)	**0.617**
	**Positive**	11 (27.5%)	29 (72.5%)	
VITAMINA.D.LEVEL	**Mean ± SD**	27.9 ± 12.5	21.9 ± 11.5	**0.064**
PT	**Mean ± SD**	13.6 ± 0.7	14.5 ± 1.7	**0.156**
INR	**Mean ± SD**	1.0 ± 0.1	1.0 ± 0.2	**0.366**
TROP.I	**Mean ± SD**	0.0 ± 0.0	0.3 ± 1.7	**0.322**
PCT	**Mean ± SD**	0.1 ± 0.1	0.4 ± 1.1	**0.539**
GLU	**Mean ± SD**	7.0 ± 1.9	9.8 ± 5.0	**0.088**

BMI: Body Mass Index; HTN: Hypertension; DM: Diabetes Mellitus; CVS: Cardiovascular Diseases; WBC: White Blood Cells; CRP: C-Reactive protein; IL6: Interleukin 6; LDH: Lactate Dehydrogenase; ALT: Alanine Transaminase; AST: Aspartate Transaminase; NLR: Neutrophil to Lymphocyte Ratio; RDW.CV: Red Cell Distribution Width; ADAMTS13: a disintegrin and metalloproteinase with thrombospondin type 1 motif, member 13; RH: Rhesus factor; PT: Prothrombin Time; INR: international normalized ratio; TROP.I: Troponin I; PCT: Procalcitonin; GLU: Glucose.

**Table 2:** Logistic regression models of the association of the demographic, clinical, and biochemical characteristics in the subgroups received tocilizumab (Unstable Severe–Early Critical–Late Critical) with COVID-19 outcome (Died–Improved).

Risk Factors		Improved	Died	OR (95% CI)	*p* Value
Tocilizumab	**Not received**	21 (100.0%)	0 (0.0%)	-	0.994
	**Received**	35 (71.4%)	14 (28.6%)	125,746,406.08 (0.00-NA)	
Unstable groups received tocilizumab	**Severe**	25 (71.4%)	4 (28.6%)	-	
	**Early Critical**	2 (5.7%)	1 (7.1%)	3.12 (0.13–41.43)	0.394
	**Late Critical**	8 (22.9%)	9 (64.3%)	7.03 (1.80–32.32)	0.007
Demographic					
Age (years)	**Mean ± SD**	47.5 ± 13.0	57.2 ± 11.5	1.06 (1.01–1.13)	0.027
Gender	**Female**	5 (14.3%)	3 (21.4%)	-	0.544
	**Male**	30 (85.7%)	11 (78.6%)	0.61 (0.13–3.37)	
Race	**Asian**	24 (68.6%)	7 (50.0%)	-	
	**Black**	1 (2.9%)	1 (7.1%)	3.43 (0.12–94.61)	0.404
	**White**	10 (28.6%)	6 (42.9%)	2.06 (0.54–7.82)	0.283
BMI	**Mean ± SD**	30.5 ± 6.2	30.1 ± 7.5	0.99 (0.89–1.09)	0.848
Clinical					
HTN	**No**	23 (71.9%)	4 (30.8%)	-	0.015
	**Yes**	9 (28.1%)	9 (69.2%)	5.75 (1.49–26.01)	
DM	**No**	21 (65.6%)	6 (46.2%)	-	0.232
	**Yes**	11 (34.4%)	7 (53.8%)	2.23 (0.60–8.58)	
CVS	**No**	26 (81.2%)	8 (72.7%)	-	0.551
	**Yes**	6 (18.8%)	3 (27.3%)	1.62 (0.29–7.80)	
Biochemical					
WBC	**Mean ± SD**	6.1 ± 2.9	8.4 ± 5.4	1.16 (0.99–1.39)	0.073
HEMOGLOBIN	**Mean ± SD**	13.6 ± 1.4	12.0 ± 2.5	0.59 (0.34–0.88)	0.026
PLATELETS	**Mean ± SD**	334.0 ± 149.6	240.9 ± 47.4	0.99 (0.99–1.00)	0.038
CRP	**Mean ± SD**	108.8 ± 82.5	202.4 ± 133.3	1.01 (1.00–1.02)	0.013
D.DIMER	**Mean ± SD**	5.5 ± 8.6	17.4 ± 8.9	1.14 (1.06–1.26)	0.002
LDH	**Mean ± SD**	439.8 ± 177.8	1145.2 ± 874.8	1.00 (1.00–1.01)	0.007
ALT	**Mean ± SD**	72.9 ± 94.6	36.8 ± 16.7	0.97 (0.93–1.00)	0.054
AST	**Mean ± SD**	64.8 ± 46.4	51.1 ± 19.3	0.99 (0.96–1.01)	0.320
CREATININE	**Mean ± SD**	0.9 ± 0.6	1.6 ± 1.3	2.59 (1.18–9.47)	0.054
NLR	**Mean ± SD**	6.0 ± 6.9	12.6 ± 8.6	1.11 (1.02–1.22)	0.020
RDW.CV	**Mean ± SD**	13.3 ± 1.6	16.5 ± 3.2	1.86 (1.32–2.90)	0.002
TROP.I	**Mean ± SD**	0.4 ± 2.0	0.0 ± 0.0	0.71 (NA-1.28)	0.735
IL6	**Mean ± SD**	451.0 ± 650.8	2109.8 ± 1319.0	1.00 (1.00–1.00)	0.045
NEUTROPHIL.COUNT	**Mean ± SD**	70.9 ± 14.8	86.3 ± 5.6	1.15 (1.06–1.30)	0.004
LYMPHOCYTE.COUNT	**Mean ± SD**	21.6 ± 12.5	9.5 ± 4.7	0.85 (0.74–0.93)	0.005
FIBRINOGEN	**Mean ± SD**	725.9 ± 149.0	837.4 ± 199.6	1.00 (1.00–1.01)	0.046
FERRITIN	**Mean ± SD**	1354.6 ± 601.7	3000.7 ± 2262.9	1.00 (1.00–1.00)	0.026
COOMB.TEST	**Negative**	18 (78.3%)	4 (40.0%)	-	0.040
	**Positive**	5 (21.7%)	6 (60.0%)	5.40 (1.13–29.64)	
BLOOD.GROUP	**A**	9 (45.0%)	3 (27.3%)	-	
	**AB**	2 (10.0%)	2 (18.2%)	3.00 (0.26–36.57)	0.361
	**B**	4 (20.0%)	1 (9.1%)	0.75 (0.03–8.30)	0.825
	**O**	5 (25.0%)	5 (45.5%)	3.00 (0.51–20.42)	0.232
RH	**Negative**	2 (9.5%)	1 (9.1%)	-	0.968
	**Positive**	19 (90.5%)	10 (90.9%)	1.05 (0.09–24.29)	
VITAMINA.D.LEVEL	**Mean ± SD**	22.4 ± 11.8	20.8 ± 11.6	0.99 (0.91–1.06)	0.724
PT	**Mean ± SD**	14.5 ± 1.9	14.3 ± 1.2	0.92 (0.51–1.48)	0.737
INR	**Mean ± SD**	1.0 ± 0.3	1.1 ± 0.1	3.42 (0.21–91.93)	0.413
PCT	**Mean ± SD**	0.2 ± 0.3	1.0 ± 1.9	4.59 (1.29–34.88)	0.065
GLU	**Mean ± SD**	9.8 ± 4.0	9.8 ± 7.0	1.00 (0.86–1.16)	0.989

BMI: Body Mass Index; HTN: Hypertension; DM: Diabetes Mellitus; CVS: Cardiovascular Diseases; WBC: White Blood Cells; CRP: C-Reactive protein; IL6: Interleukin 6; LDH: Lactate Dehydrogenase; ALT: Alanine Transaminase; AST: Aspartate Transaminase; NLR: Neutrophil to Lymphocyte Ratio; RDW.CV: Red Cell Distribution Width; RH: Rhesus factor; PT: Prothrombin Time; INR: international normalized ratio; TROP.I: Troponin I; PCT: Procalcitonin; GLU: Glucose.

**Table 3:** The potential effect of co-management of vitamin D, anticoagulants, antivirals, and steroids with tocilizumab on COVID-19 outcome.

Co-Management		Died	Improved	*p* Value
Vitamin D	No (N = 30/41)	12 (40.0%)	18 (60.0%)	0.07
	Usual Treatment Dose (N = 12/20)	2 (16.7%)	10 (83.3%)	
	High Dose (N = 7/9)	0 (0.0%)	7 (100.0%)	
Anticoagulants	Non-therapeutic Dose (N = 10/12)	10 (100.0%)	0 (0.0%)	<0.001
	Therapeutic Dose (N = 39/54)	4 (10.3%)	35 (89.7%)	
Steroids	No (N = 46/63)	12 (26.1%)	34 (73.9%)	0.193
	Yes (N = 3/7)	2 (66.7%)	1 (33.3%)	
Antivirals	HCQ/AVIGAN (N = 24/38)	7 (29.2%)	17 (70.8%)	0.551
	HCQ/AVIGAN/KALETRA (N = 7/8)	2 (28.6%)	5 (71.4%)	
	HCQ/AVIGAN/KALETRA/AZI (N = 2/3)	0 (0.0%)	2 (100.0%)	
	HCQ/AZI (N = 6/7)	0 (0.0%)	6 (100.0%)	
	HCQ/AZI/AVIGAN (N = 11/14)	4 (36.4%)	7 (63.6%)	
	HCQ ALONE (N = 0/1)	0 (0.0%)	0 (0.0%)	

AZI: Azithromycin; HCQ: Hydroxychloroquine; AVIGAN: Favipiravir; KALETRA: Lopinavir-Ritonavir.

**Table 4:** The association between each secondary infection and adverse events post-tocilizumab with the clinical condition.

AE and Secondary Infection		Severe	Early Critical	Late Critical	*p* Value
Adverse events	**Grade 1**	10 (90.9%)	0 (0.0%)	1 (9.1%)	0.006
	**Grade 2**	2 (40.0%)	0 (0.0%)	3 (60.0%)	
	**Grade 3**	2 (18.2%)	1 (9.1%)	8 (72.7%)	
	**Grade 4**	2 (66.7%)	0 (0.0%)	1 (33.3%)	
Secondary infections	**Bacterial**	0 (0.0%)	1 (20.0%)	4 (80.0%)	0.024
	**Fungal**	2 (66.7%)	0 (0.0%)	1 (33.3%)	
	**No**	27 (65.9%)	2 (4.9%)	12 (29.3%)	

Grades of AE: Grade 1 Mild AE, Grade 2 Moderate AE, Grade 3 Severe AE, and Grade 4 Life-threatening or disabling AE.

## Data Availability

Data can be available upon request from the first and corresponding author.
